# Functionalized Nanomaterials as Tailored Theranostic Agents in Brain Imaging

**DOI:** 10.3390/nano12010018

**Published:** 2021-12-22

**Authors:** Ramar Thangam, Ramasamy Paulmurugan, Heemin Kang

**Affiliations:** 1 Department of Materials Science and Engineering, College of Engineering, Korea University, Seoul 02841, Korea; 2 Institute for High Technology Materials and Devices, Korea University, Seoul 02841, Korea; 3 Molecular Imaging Program at Stanford (MIPS), Department of Radiology, Stanford University School of Medicine, Stanford University, Palo Alto, CA 94304, USA; paulmur8@stanford.edu; 4 Molecular Imaging Program at Stanford, Canary Center at Stanford for Cancer Early Detection, Stanford University School of Medicine, Stanford University, Palo Alto, CA 94304, USA; 5 Department of Biomicrosystem Technology, College of Engineering, Korea University, Seoul 02841, Korea

**Keywords:** functionalized nanomaterials, contrast agents, imaging, delivery, theranostics

## Abstract

Functionalized nanomaterials of various categories are essential for developing cancer nano-theranostics for brain diseases; however, some limitations exist in their effectiveness and clinical translation, such as toxicity, limited tumor penetration, and inability to cross blood–brain and blood-tumor barriers. Metal nanomaterials with functional fluorescent tags possess unique properties in improving their functional properties, including surface plasmon resonance (SPR), superparamagnetism, and photo/bioluminescence, which facilitates imaging applications in addition to their deliveries. Moreover, these multifunctional nanomaterials could be synthesized through various chemical modifications on their physical surfaces via attaching targeting peptides, fluorophores, and quantum dots (QD), which could improve the application of these nanomaterials by facilitating theranostic modalities. In addition to their inherent CT (Computed Tomography), MRI (Magnetic Resonance Imaging), PAI (Photo-acoustic imaging), and X-ray contrast imaging, various multifunctional nanoparticles with imaging probes serve as brain-targeted imaging candidates in several imaging modalities. The primary criteria of these functional nanomaterials for translational application to the brain must be zero toxicity. Moreover, the beneficial aspects of nano-theranostics of nanoparticles are their multifunctional systems proportioned towards personalized disease management via comprising diagnostic and therapeutic abilities in a single biodegradable nanomaterial. This review highlights the emerging aspects of engineered nanomaterials to reach and deliver therapeutics to the brain and how to improve this by adopting the imaging modalities for theranostic applications.

## 1. Introduction

**Figure 1:**
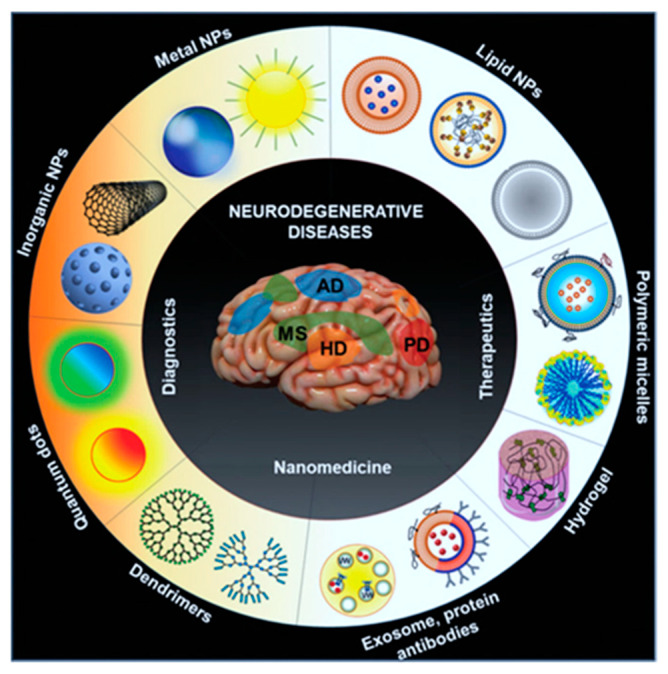
Scheme identifies the emerging different kinds of nanomaterial formulations attempted for the improved drug delivery approaches in neurological diseases. Reprinted with permission from Ref. [8]. Copyright 2021 John Wiley and Sons.

The remarkable developments in multimodal molecular imaging methods using various functional nanomaterials have led to the translation of many novel materials into the clinic. These nanomaterials are established to confront the crucial problems encountered by diagnostic imaging techniques [1,2]. Theranostic imaging adopting nanomaterials presents significant improvements over the traditional approaches via increasing blood circulation times, enhanced diagnostic specificity, and organ-specific delivery [3]. Theranostic imaging modalities improve the understanding of various biological processes via direct observation of available events in real-time. In recent years, growing interests in image-guided theranostics have elevated the researchers’ directions to study and understand the mechanistic aspects of multiple disease-related signaling to recognize and enable easy and early diagnosis [4]. This further helped them to identify the complex neural networks in the brain process of cognitive therapies. Imaging techniques, such as MRI, fluorescence, bioluminescence, FRET (Förster (or Fluorescence) Resonance Energy Transfer), BRET (Bioluminescence resonance energy transfer), US (Ultra-sound), and PAI, provide necessary information about brain functions at anatomical, cellular, and molecular levels [5,6]. Furthermore, the image-guided biological studies facilitate researchers to better understand the detailed biochemical processes involved in metabolic and functional physiological events (the macromolecular interaction in cells at healthy or diseased states) and translate them into pathologic functions associated with the disease to establish independent research directions [7]. Abilities of engineered nanomaterials generated in theranostics ensure the dual capacity of therapeutic delivery [8] and diagnosis to a preponderance of personalized medicine in clinical conditions to target different neurological diseases (Figure 1). In spite of all these, the nanomaterials produce the imaging signals of delivered molecules which could carry the essential features of molecular imaging and which further translate the imaging signals towards image acquisition. Several nanostructured materials [9] with magnetic properties [10,11] are emerging due to their functional properties towards biomedical applications with reduced toxicity. However, these nanostructures could be regulated via external stimuli-based magnetic fields that could regulate a variety of tissue modulatory potentials in vivo [12,13,14]. This functionalization could improve the target functions, biocompatibility, and surface area modifications in biological and clinical translations [15,16,17]. One of the major criteria for any molecular imaging in brain theranostics would be the target-specificity of the delivery method with improved biocompatibility at the delivered site without much toxicity. The principal imaging associated factors, such as high sensitivity, non-invasive imaging detection systems, signal penetration without attenuation, and temporal and spatial resolution, are the significant factors that are critical for ensuring the clinical potential of the developed methods [18]. These factors should be carefully included while making the functional nanoparticles for imaging applications of the brain. In addition, these nanoparticles should possess properties that enhance image quality, contrast, and targeting features during their modifications, which could play prominent roles in target-specific molecular screening [19,20].

Brain with its complex neural network is difficult to study using various biochemical techniques. Non-invasive molecular imaging techniques with the potential to monitor dynamic physiological events in the brain could be an effective strategy for studying the complex processes of the nervous system [21]. One of the significant breakthroughs in the longitudinal image monitoring of animals is molecular imaging, which allows researchers to observe biological events at physiological conditions without the need for sacrificing the animals [22]. Gold nanoparticles with various surface modifications using imaging probes/candidates have allowed X-ray contrast imaging in disease diagnosis, including cancer. These innovative strategies might be applicable to detect tiny tumors in multiple organs, including the brain, for glioma, when they are a few-millimeter in size in vivo [23,24]. In addition, the enzyme-modified functional gold nanoparticles systems were applied to make nanoparticles with the property of self-assembly and disassembly in vivo at the delivered target sites [25,26,27]. Moreover, novel nanomaterials functionalized with fluorescent probes can be used for image-guided disease theranostics of the brain, while the coupled chemical agents functionalized at the surface of the nanoparticles could enhance the targeted delivery and therapy during theranostic applications [28,29]. These contrast agents further help the researchers to get more detailed information via delivered functional nanomaterial systems to understand the biological systems and their interrelationship much better than the conventional methods [30,31].

**Figure 2:**
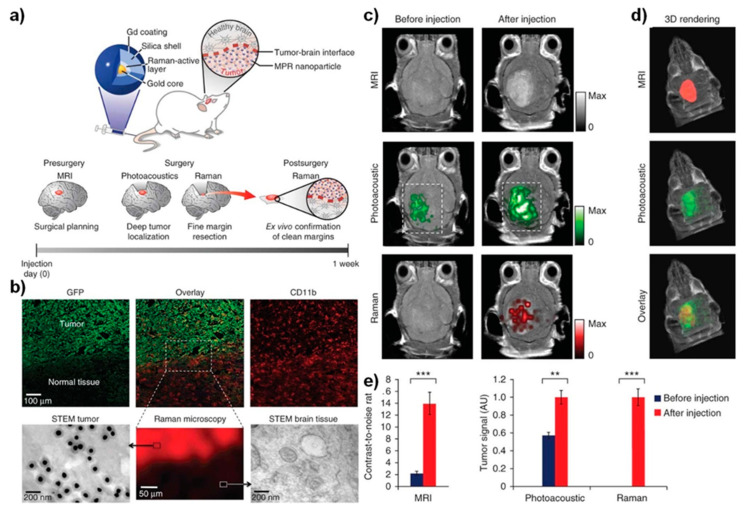
Triple-modality nanoparticle delivery and imaging concept to the brain tumor model. (**a**) Delivery of nanoparticles circulates in the bloodstream; they diffuse through the disrupted blood–brain barrier and are then sequestered and retained by the tumor; upon employing photoacoustic imaging, the high resolution, and deep tissue penetration guide tumor resection intraoperatively in the surgical room. Following imaging strategies of the brain specimen can subsequently be examined as an imaging probe ex vivo to validate clear tumor margins. (**b**) Immunohistochemistry of the tissue sections from the margin of the brain tumor stained for glial cells under confocal laser scanning microscopy. Scanning transmission electron microscope (STEM) images validated the presence of delivered nanoparticles in the brain tissue, whereas no such nanoparticles were seen in the healthy brain tissue. (**c**) Two-dimensional axial MRI, Photoacoustic, and Raman images; (**d**) three-dimensional (3D) rendering of magnetic resonance images with the tumor segmented overlay of the three-dimensional photoacoustic images. (**e**) Corresponding quantitative signals of the nanoparticles from images shown in (**c**,**d**). Shown data represents mean ± S.E.M; *** *p* < 0.001, ** *p* < 0.01. Reprinted with permission from Ref. [43].Copyright 2021 Springer Nature.

In view of the perspective, the blood–brain barrier (BBB), which interfaces between the central nervous system (CNS) and the vascular compartment, can block the delivery of administered nanoparticles circulating in the body to the brain’s extravascular cells. BBB is critical in protecting brain cells from the vascular contents to maintain homeostasis; in contrast, it represents an intense challenge in drug delivery applications. The permeability modulations of BBB can be graded by light-laser intensity [32]. The permeability of BBB is entirely reversible and involves increased paracellular diffusion or opening at the delivered sites without leading to a significant disruption in the structure of neurovascular units of the brain. This strategy allows the delivery and entry of multiple therapeutic agents, such as immunoglobulins and viral gene therapy vectors, cargo-laden liposomes, functionalized nanomaterials, peptide conjugates, and nanomaterials with small molecules [33]. We anticipate this theranostic nanotechnology development might be helpful in tissue regions accessible to novel imaging applications and open novel venues in screening and therapeutic interventions in CNS diseases. Thus, the recent emergence of nanomaterial systems could be applied for disease or cellular pathway imaging at the molecular level by linking with theranostic imaging fields via providing potentials to grow molecular imaging field both in vitro and in vivo applications [34,35,36]. Furthermore, various imaging methods, such as optical/magnetic resonance, have been achieved via developing shape-tuned nanomaterials, such as nanospheres, nanorods, nanocoils, and nanoclusters, to test under various physiological conditions [9,14,37,38]. Moreover, the currently used nanoparticles for MRI imaging have broader magnetic properties, and most of them are paramagnetic or super-paramagnetic in nature [39,40]. In addition, the other oxidized forms of metal nanomaterials (i.e., MnO and SPIONs, etc.) carry the MR-imaging (Figure 2) features have been applied in brain theranostics [41,42,43]. There has been the emergence of gold nanoparticles, nanorods, carbon nanoparticles or nanotubes, and graphene oxide nanomaterials, which are being translated to photoacoustic and other functional imaging modalities in neurological disorders, including brain cancers.

**Figure 3:**
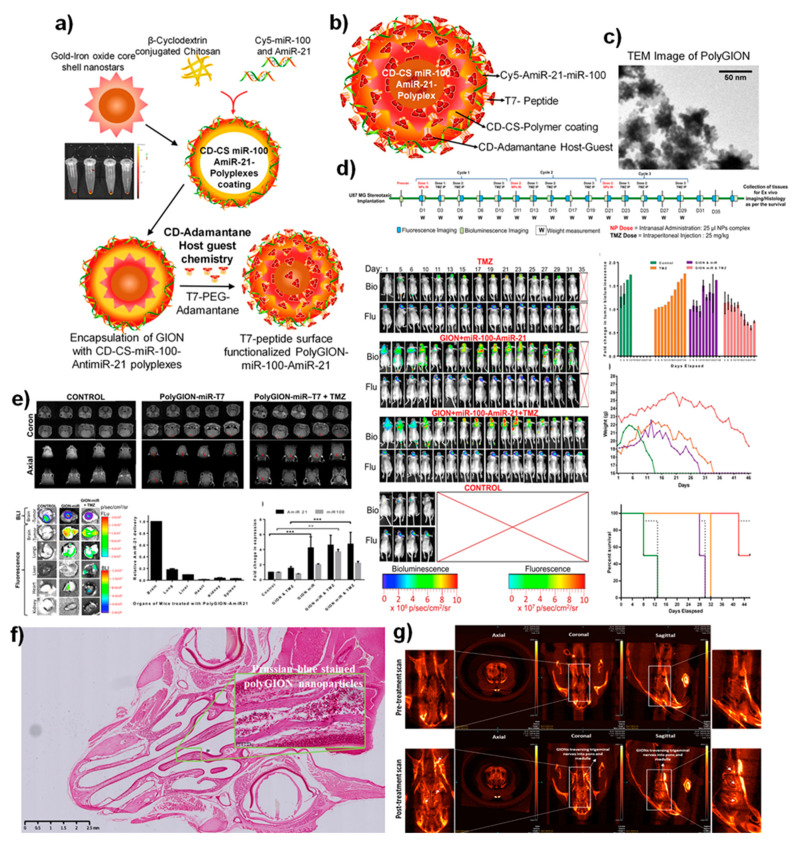
(**a**) Schematic illustration of the synthesis of Poly-gold-iron oxide nanoparticles (polyGIONs) system and in vitro fluorescence images of Cy5 labeled miR-100 and antimiR-21 loaded cyclodextrin-chitosan (CD-CS) hybrid polymer complexes. (**b**) Schematic of the as-prepared polyGION nanoparticle structure and the associated compositions. (**c**) TEM micrograph of GIONs. (**d**) In vivo treatment flow chart of the therapeutic design and imaging timelines; fluorescence (Cy5-miRNA loaded nanoparticles) and bioluminescence (FLuc-EGFP expressing glioblastoma model); quantitative measurements for the tumor bioluminescence measured concerning treatment duration; mice body weight profiles over the treatment duration and their survival curve indicates the intranasally delivered nanoparticles towards the theranostic efficacy. (**e**) 3T MRI scanning (coronal and axial) of the polyGIONs-miRNAs treated mice brain imaging; biodistribution; ex vivo fluorescence imaging, and qRT-PCR of antimiR-21 and miR-100 expression levels. Reprinted (adapted) with permission from Reference [51]. (**f**) H&E-stained histological image shows the nasal epithelium, followed by iron-specific Prussian blue staining (inset figure) to trace the accumulation of polyGION nanoparticles in mice intranasal cavities. (**g**) microCT imaging of mice head scan shows the non-treated (control) and T7-polyGION-CD-CS NPs administered in vivo. Corresponding microCT scan images depict the migration of IN administered T7-polyGION-CD-CS NPs nanoparticles movements through the olfactory nerve pathway into the olfactory bulb and passing into trigeminal nerve pathway, thereby entering the pons and medulla of the mice brain. Shown data represents mean ± S.E.M; *** *p* < 0.001, ** *p* < 0.01. Adapted with permission from Ref. [51], with permission. Copyright 2021 Elsevier.

To further envisage the importance of nanomaterials in brain theranostics, functional nanomaterials with the widespread application have been developed with significant roles in the field of emerging nano-theranostics of molecular imaging. Nanotechnology is an engineering discipline that comprises the characterization and application of nanoscale (1–999 nm) materials in a single structural dimension for disease diagnosis applications. Most of the engineered nanomaterials are designed to display various functional features, such as high sensitivity, selectivity, and tunable properties, that are absent, or their properties vary from other bulk materials [44,45]. In addition, these nanoscale materials can be modified to possess novel features, such as optical, magnetic, structural, and electronic properties, which are not originally present in the bulked materials. These utilizations of nanomaterials are attracting their applications in various aspects of drug delivery, molecular diagnosis, theranostics, and improved cancer therapies, including brain cancer or other neurological disorders, such as Alzheimer’s and Parkinson diseases. However, before we apply these nanomaterials for brain theranostics of nanotechnology in novel drug delivery and imaging, there must be several aspects that should be considered for better outcomes, which include improved biocompatibility, less or no toxicity, non-agglomeration in the body upon injection, longer half-life, and in vivo stability and improved target specificity with enhanced imaging qualities at the target sites [46]. As shown in Table 1, several forms of nanomaterials have been engineered and applied for the brain targeting functions with improved therapeutic outcomes. In addition to that, Zhang et.al. developed an engineered nanomaterial platform to act as disease contrast agents in brain imaging. These materials showed less toxicity and a prolonged circulation time upon delivery [47]. In addition, the potentialities of nanomaterials associated toxicities could be escaped via certain structural modifications in the shape or properties, whereas the nanomaterials controlled to show slow and sustained release at the delivery site of the brain upon linked using targeting receptors/ligands involved to suppress the associated toxicities in vitro and in vivo [48,49,50]. Recently, Sukumar et.al. developed a multifunctional nanosystem using gold iron oxide nanoparticles (GIONs) conjugated with receptor targeting peptides with less toxicity for targeting glioblastoma via intranasal administration to overcome BBB [51]. This smart construct delivered the small therapeutic microRNAs that altered gene expression while facilitating contrast CT and MR-imaging of the glioma cells. The delivered microRNA further sensitized the tumor cells to the delivered Temozolomide (TMZ) anticancer agent in vivo (Figure 3). Similarly, Zhou et.al. developed a non-toxic nanoimaging material that specifically measured angiogenesis in glioblastoma [52]. Thus, the emerging nanomaterials platforms to brain theranostics would emphasize this field upon using their distinctive characteristics, further supporting them for biomedical imaging modalities, including drug delivery and therapeutics [53].

Likewise, the intensive role of intravenous injectable nanomaterials also possesses tremendous therapeutic and diagnostic efforts to act as a tool for theranostic nanomaterials in the treatments of brain-related diseases [77,78]. The physiological barriers, such as BBB-related events of therapeutic blockings, could be improved via a variety of target-specific functional processes of the nanomaterials [79]. The injectable nanoformulations of multifunctional nanomaterial categories possessing the optical, thermal, and magnetic fields would be a promising strategy to improve the theranostic modalities in brain disease management. Overcoming these internal barriers in nanomaterial administration could serve as a nanomaterial for multifunctional features towards better insights into the innovative strategic developmental aspects of most favorable and feasible materials systems towards clinical applications [80].

## 2. Nanomaterials Improving Theranostic Imaging Modalities

**Figure 4:**
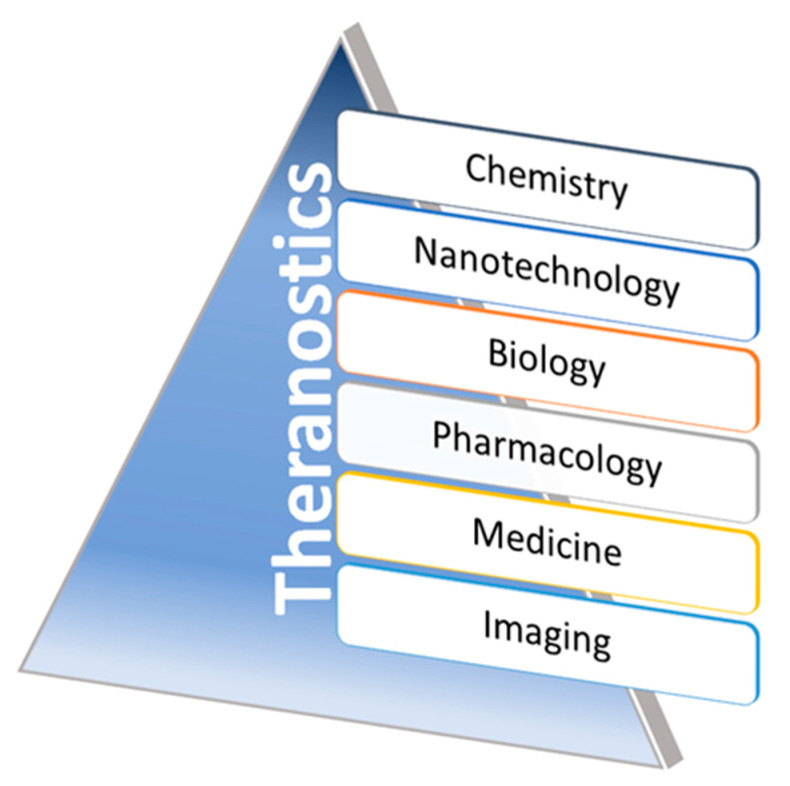
Schematic representations of the growing contributing fields of theranostics. Representative illustration showing the contributing interdisciplinary fields of nanomaterials associated with theranostics. Via adopting these multidisciplinary fields, the innovative nanomaterial formulations aim to involve disease monitoring, diagnosis, and therapy through the researcher’s intersections of multiple scientific fields.

Theranostic nanoparticles in molecular imaging significantly impact non-invasive strategies to understand biological and biochemical events in intact cells within living subjects. It plays a prominent role in disease diagnosis and therapeutic monitoring outcomes in vivo [27]. The theranostic application of nanomaterials can be classified into morphological and functional imaging based on their roles in image contrast abilities during applicable imaging methods. A wide range of multifunctional nanoparticles have been extensively proven for their properties as an agent for both therapeutic and diagnostic applications (theranostics). Promoting newer research directions are shown to explore those novel materials function in relevant animal disease models via improving their qualities towards clinical translations—recent approaches in non-invasive disease monitoring, biomarkers, and therapeutic drug deliveries are under investigation in advanced theranostics. In addition, some of the biomaterials, including magnetic NPs, QDs (Quantum dots), UCNPs (Upconverting nanoparticles), SLNs (silica nanomaterials), carbon nanoparticles, and organic dye coupled materials, have shown a significant role in theranostics with wide ranges of clinical translations. Variations in size and surface changes could modulate biocompatibility and interactions of these nanomaterials with target tissues. Hence, developing the contracted interest for improved disease monitoring/detections with improved chemotherapies along with clinically translatable innovative nanomaterials can be a significant driving force for theranostic agent research in the near future. The advancements made in, and the tie between, interdisciplinary scientific disciplines, such as nanotechnology, biology, pharmacology, chemistry, medicine, and imaging fields involving developing theranostics, have been significantly designed and evaluated over the past few years in clinical conditions, for growing nano-theranostics (Figure 4).

The development of novel nanoparticles consisting of both diagnostic and therapeutic components has increased over the past decade. These theranostic nanoparticles have been tailored toward one or more types of imaging modalities. They have been developed as imaging probes in optical imaging, magnetic resonance imaging (MRI), photoacoustic imaging (PAI), computed tomography (CT), and nuclear imaging comprising both single-photon computed tomography (SPECT) and positron emission tomography (PET). Here, we focused on the brain theranostic nanoparticles capable of both delivering therapy and self-reporting/tracking disease through imaging. Generally, imaging modalities, such as optical imaging, SPECT, and PET, are performed using a broad range of probes with high sensitivity [81]. The other primary imaging modalities, CT and MRI, are also reduced by image contrast properties during the probe conjugation or the tailored agents in the surface modification to impact resolution and sensitivity further. The growing interest in applying CQD (carbon quantum dots) with functionalized nanomaterials attention to various brain-related drug delivery approaches is emerging towards the clinical need of the situation. Utilizing these types of nanomaterials in combination with external stimuli, such as light or photoacoustic waves, at the delivery site, could efficiently modulate the functional properties of chemotherapeutic agents with diagnostic abilities [82,83]. The broader utilization of CQD in clinical applications is increasing because of their role in various aspects, including imaging and drug/gene delivery with therapeutics. The use of CQD in drug delivery across the BBB was achieved via nanoparticles after several functionalization processes in their structure that further internalized to the glioma cells, thereby envisaging their potentials in theranostic modalities in vitro and in vivo [84,85,86]. Thus, the novel nanomaterials in various categories could overcome these limitations by enhancing the tissue penetration, biodistribution at the desired sites, and the target specificity while adopting them in molecular imaging application [87]. Moreover, the nanomaterials are economically affordable and accurately deliverable for molecular-level quantitative imaging information towards translational approaches. Several interdisciplinary areas that range from novel targeting strategies, combination therapies, and unique imaging prospects via multiple multidisciplinary fields are emerging in recent decades to enhance theranostics (Figure 4). Despite significant progresses in developing MRI-targeted nanotheranostic platforms and their undeniable potential in predictive, preventive, and personalized medicine, gaps in knowledge continue to hinder their translations from bench to bedside, and a few nanotheranostic systems have undergone clinical trials [88]. This can be due to several factors, including the complexity of the developed hybrid nanosystems, difficulty in predicting their complex effects and interactions with biological systems, species-dependent immune responses and toxicity profiles, difficulty in controlling the pharmacokinetics and biodistribution properties, premature release of the therapeutic cargos in blood and healthy tissues, toxicity concerns, and the significant differences between animal models and humans. Recent research has focused on using imaging data to understand better the interactions between nanoparticles and biological systems to optimize tumor targeting and biodistribution [89].

In recent years, the growing interest in multimodal theranostics has been getting broader to overcome most contrast-associated limitations; nanomaterials with image contrast functions can provide more optimal materials to study the physiological and anatomical data retrieval in disease diagnosis with treatments (theragnosis). Hence, molecular engineers and nanotechnologists are trying to improve the existing nanomaterials contrast in disease detections in several human diseases, including neurological disorders [90]. Furthermore, various research groups work towards brain targeting and imaging with multifunctional nanomaterials strategies for suitable optical and MR-imaging modalities [24,91,92]. In practice, the multimodal targeting of tumors in the brains or the microenvironments could reach via engineered nanomaterials to enhance their theranostic functions (Table 1). In addition to integrating the disparity of dosage requirement between diagnostic and therapeutic entities within a single nanoparticle platform, the emerging tailored theranostic technologies could be applied to optimize the differences by estimating their circulation times that will further necessitate to realize the potentialities.

## 3. Nanoparticle-Based Intranasal Delivery of Therapeutics to Target Cancers in the Brain

Nanomaterials and their corresponding formulations have been widely applied for various biomedical applications since they possess different physical and chemical properties. In addition, the nanomaterials possess a higher surface area, allowing them to carry or conjugate various organic moieties, such as peptides, drugs, and polymeric substances, in sufficient quantities. In this regard, glioblastoma is the deadliest and recurrent form of malignancy among various cancer types, which mainly grows and massively infiltrates the surrounding brain parenchyma. The current clinical treatments are surgical resection of the tumor, followed by chemo- and radiotherapies. However, these approaches are still insufficient to completely eradicate the tumors or reduce recurrences to achieve disease-free survival [93]. Emerging trends in nasal delivery of target-specific agents had been proposed to carry a variety of non-invasive strategies to directly reach the brain microenvironments via bypassing the various barriers, such as the blood–brain barrier [94]. Moreover, this is emerging as a novel therapeutic administrative strategy for delivering pharmacologically active moieties, imaging agents, and nanomaterials for functional mechanistic activations. This approach is currently a prominent and emerging method for glioma treatments of the brain than other modalities because they offer potential drug delivery towards other neurological disorders in the brain [95]. However, the treatment choices depend on various aspects, such as type of glioma, location, stage, and their size, in the patient survival rate, which could predict the intranasal drug delivery and treatments. Past reports found that tumors pre-sensitized with therapeutic miRNAs could show rapid reduction in tumor volumes upon chemotherapy and facilitate imaging functions upon treatment with engineered nanomaterials for intranasal delivery [51,68]. Novel approaches to bypass the physical barriers and challenges to cognitive disease treatments are always dependent on finding alternative ways for the direct route to the brain. The better drug distribution in the neurological site or microenvironments could be achieved through intraventricular aspects as compared to the other methods in intraventricular, intrathecal, or nasal administrations. Though a better standard is needed to improve this strategy to control and monitor the deliverable implant and drug release controlling modalities during administration [96].

CNS diseases represent the most significant and rapidly growing research fields with unmet clinical needs. Hence, nanotechnology plays an instrumental role in the revolutionary development of brain-specific drug development, delivery, imaging, and diagnosis. With the aid of nanoparticles of high specificity and multifunctionality, such as dendrimers, quantum dots, therapeutic drugs/small RNAs, imaging agents, and other diagnostic molecules, can be delivered to the brain across the blood–brain barrier (BBB), enabling considerable progress in the understanding, diagnosis, and treatment of CNS diseases [80]. Nanoparticles used in the CNS for drug delivery, imaging, and diagnosis are well demonstrated. Similarly, the administration routes, toxicity, and mechanism to facilitate or cross the BBB have also been demonstrated. To date, no single delivery strategy can be able to provide a definitive solution to all the problems associated with brain drug delivery. Developing innovative administration routes is as important as developing new delivery systems. It is desired to create an effective brain drug delivery system and a non-invasive, safe, low-cost way to administer it with ease of application, increasing treatment efficacy and patient compliance, and reducing societal service burden [97,98]. A variety of nanoparticles have been developed and engineered for specific applications in the brain. With the aid of nanomaterials of high specificity and multifunctionality, therapeutic, imaging, and diagnostic molecules can be delivered to the brain across the BBB enabling considerable progress in the fundamental understanding, diagnosis, and treatment of brain disorders and diseases. Because of the inherent complexity of the brain, the safety concerns of nanomaterials for nanoparticle-mediated technologies have shown great promise, which should be further scrutinized prior to their clinical applications [99].

Intranasal (IN) delivery is a rapidly developing area for therapies with great potential for treating brain diseases (Table 1). Moreover, in vivo imaging is becoming an essential part of therapy assessment, both preclinically in animals and clinically in human translational applications. IN drug delivery is an alternative to systemic administration, which uses the direct anatomic pathway between the olfactory/trigeminal neuroepithelium of the nasal mucosa and the brain. Several drugs have already been approved for IN delivery applications, and a few others are under development and testing. To better understand the delivery and therapeutic action, several imaging modalities are being used with the potential in vivo imaging for both humans and animals, including MRI, PET, SPECT, and CT imaging. Additionally, in vivo optical imaging modalities, including bioluminescence and fluorescence, have been extensively used in pre-clinical settings [100]. We outline the growing interest of imaging modalities in brain imaging, how it is being utilized, and its strengths and weaknesses, specifically in the context of IN delivery of therapeutics, in this section.

**Figure 5:**
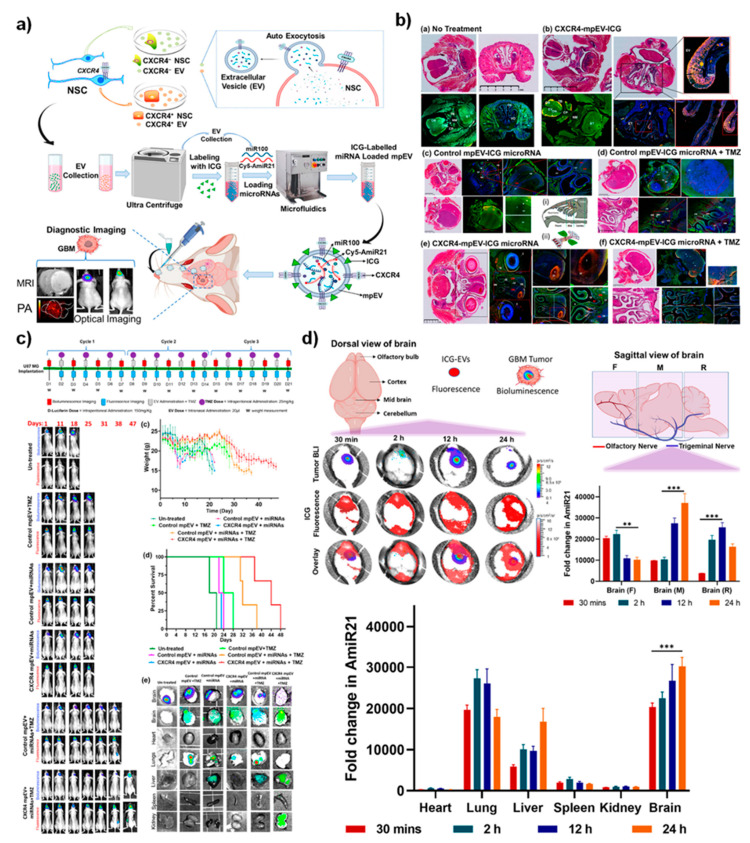
(**a**) Schematic illustration explains the microfluidic reconstruction of miRNA-loaded extracellular vesicles (EVs) for intranasal delivery towards the enhancements of theranostic imaging in glioblastoma tumor-bearing mice model. (**b**) H&E and confocal laser scanning immunohistochemical images of cranial sections of animals treated with IN delivered EVs and (**c**) corresponding therapeutic monitoring of IN delivered EVs associated targeted nanomaterial platform with respective control groups, in co-treatment with temozolomide in vivo. (**d**) Diagrammatic and sagittal views of the brain delivered with EVs associated nanomaterials by ex vivo bioluminescence and fluorescence imaging showing intranasal administration at varied time-points in vivo. Shown data represents mean ± S.E.M; *** *p* < 0.001, ** *p* < 0.01. Reprinted with permission from Ref. [68]. Copyright 2021 American Chemical Society.

As stated above, the nasal cavity is well-suited for therapeutics delivery to the brain. The nasal mucosa has a high relative permeability, thin endothelial membrane, and good surface area for absorption of small molecules and macromolecules, such as proteins, peptides, nucleic acids, viruses, and even stem cells. In particular, nose-to-brain therapy delivery has garnered high interest given the high failure rate of drugs that cannot bypass the BBB. Using nanomaterial systems, several therapeutic molecules are undergoing experimental evaluation using the IN route in animal models. Novel strategies in IN delivery that enable water-soluble molecules to cross the BBB have been developed and reviewed [35,101,102,103]. These efforts include the use of receptor-mediated transport systems, peptidomimetics, monoclonal antibodies, and particulate drug carrier systems [17,18]. While these efforts are showing some promise, the ability to deliver biological macromolecules directly to the CSF (cerebrospinal fluid), which bathes the brain and spinal cord, is currently one of the most promising approaches to surmount existing delivery barriers. Over the past decade, nanotechnology and nanomedicine have been considered promising therapeutic tools for neurodegenerative diseases. Among them, organic, inorganic, and polymer nanomaterials have been shown to possess a wide applicability for relieving neural disorder symptoms via neural interfaces, neuronal differentiation, and neural stimulation via enhancing biocompatibility and reduced cytotoxicity. However, one of the limitations for brain-targeting drug delivery systems or treatment methods is the restricted entry of active compounds to the central nervous system via the BBB. Most of the current nanomedical approaches have been limited to directly targeting brain tissue through intracranial injections. Thus, minimally invasive techniques need to be developed to enhance nanoparticle delivery, including nanomedicine. To address this issue, Wang et.al. recently showed that the extracellular vesicles (EVs) of various cells could be used as a potential carrier to load therapeutic miRNAs against glioblastoma in a microfluidic platform for potential IN delivery [68]. This study proved that the delivery of EVs targeting CXCR4-SDF1α receptor axis in the orthotopic glioblastoma models enhances the delivery of loaded miRNAs via bypassing the BBBs of the mice intracranial compartments in vivo. In Figure 5, the study proposed the principles and way of intranasal (IN) administration of prepared EVs in mice. Delivery of therapeutic EV combinations enhanced localization and theranostic imaging potential (ICG dye labeled EVs) in brain microenvironments. From these outcomes, the delivered EVs associated platform showed the improved targeted delivery and therapeutic advancements in the glioblastoma treatments during the co-administration of temozolomide, thereby enhancing the theranostic values of the delivered nanomaterials in clinical advances of brain tumor management.

The acute or chronic drug treatments for different brain disorders, including neurodegenerative diseases and cancers, are challenging in several aspects. Limited bioavailability and exposure of the oral drugs to the brain, the quick metabolic process, and less toxicity, higher dosages, and increased costs of the medicines are directed the current research towards the alternative approaches of the brain associated diseases. Low brain penetration of the compounds has to overcome the BBB, protecting the brain against xenobiotics. Intranasal drug administration is one of the promising options to bypass the BBB, which reduces the drugs’ systemic adverse effects and lowers the doses to be administered [104]. Furthermore, the nasal route medications usually have higher bioavailability, fewer side effects, and higher brain exposure at a smaller dose than the oral drugs. One of the factors that needs to be respected is the role of transporters of drug influx and efflux transporters; hence, new drugs must be studied concerning their ability to be removed by these efflux transporters or if it is possible to use specific inhibitors that may improve the therapeutic and imaging efficacy by altering the pharmacokinetic profile of drugs in the brain following intranasal or intraolfactory administration of nanomaterials. Taken together, research and development in the field of intranasal or intraolfactory drug administration by nanomaterials is a rapidly growing area in brain drug delivery [68,75,105]. In addition, newer nanomaterials in theranostics will demand innovation in therapeutic targets in the brain, where more specific delivery systems need to be developed.

Much of the efforts to improve the quality of patient care has been focused on improving the targeting ability of cancer nano-theranostics via exploiting the molecular signatures of cancer cells to deliver the optimum drug dosage to tumors without harming healthy cells, which is vital in brain microenvironments. Nanotechnology offers the opportunities to combine this drug targeting with biomedical imaging, specifically MRI with its high spatial resolution, and other treatment modalities to overcome the challenges of cancer diagnosis and therapy. The recent developments of multifunctional, cancer-targeted nanotheranostics comprised of targeting molecules, imaging agents, and therapeutic agents, for MRI-based diagnosis and treatment of tumors and other brain-related disorders in vivo support this outcome [106]. Another challenge for clinical translation of novel contrast agents is the high dose necessary to achieve the desired diagnostic and therapeutic response, creating safety and toxicity issues. In addition, the low utilization rate and poor market performance of prior FDA-approved, nanoparticle-based MRI contrast agents, due to the availability of better alternatives, has resulted in diminished interest and difficulty in receiving significant investments required for the development of new agents [22]. Therefore, a prospective contrast agent should have a large market size and provide beneficial diagnostic information to justify the high cost associated with its development and use. Incorporating therapeutic capabilities in these agents helps justify the high cost of development as therapeutics have a significantly larger market size than pure diagnostic agents. However, an “all in one” nano-theranostic platform requires complex synthesis routes and often shows a premature release of cargo, resulting in severe side effects [107]. In the end, clinical translation hinges on proving enhanced efficacy over existing nanomaterials in theranostics and demonstrating sufficient biocompatibility. However, proper treatment planning may envisage considering the strengths and limitations of each modality and the physiology, distribution, and type of brain-related diseases, including cancers and neurological disorders to be treated.

The improvements in multifunctional nanocarriers possess significant advancements in targeted drug delivery and in vivo imaging; this may bring many essential treatments for neurodegenerative diseases. The advances in nanotechnology have revolutionized the successful transfer of drugs across the brain. The drugs are either encapsulated or attached to the surface of the nanomaterials through a specific modification process that could promote the theranostic modalities to the delivered sites. However, significant in vivo studies are required to track the causes of the disease and improve the drug-delivering and imaging strategies [108]. As described in Table 1, there have been a variety of intranasally deliverable nanomaterials formulations attempted for treating brain disorders. As well, a few of them had been utilized with imaging agents during delivery via using their imaging tags or contrast behaviors in vivo [36]. Hence, therapeutic administration by intranasal delivery routes appears to be an emerging newer method to deliver agents to the neurological sites of various disease microenvironments. Despite these, there are certain limitations with intranasal drug deliveries, which include the enzymatic degradations, poor bioavailability of therapeutic peptides, proteins, the mucociliary transport associated intranasal high clearance, and other anatomical associated barriers in various aspects of volumes, surface area, mucus barriers, etc. [109]. Thus, emerging potentialities and theranostic modalities to innovative therapies of neurological disorders via various delivery systems are under investigation. The growing interest in functionalized nanomaterials platforms offers several advantages over conventional therapeutic administrations across blood–brain barriers in some instances [110]. Utilizing the functional nanomaterials platforms can also improve the therapeutic aspects in various forms, such as less administration frequency, controlled drug release, site-specific delivery, stability in microenvironments, etc. [111]. The functional nanocarriers could also be tagged with imaging probes or moieties by a surface modification to enhance the contrast behavior in multimodal imaging [112]. In addition, long-term investigations are necessary to demonstrate the promising results in better distribution clearly and improved therapeutics of intranasal delivery via applying the intranasal BBB strategies. This may possibly prove the novel therapeutic innovations in brain-related disorders with multimodal imaging applications.

## 4. Theranostic Modalities with Functionalized Nanoparticles for Brain Diseases

The magnetic resonance imaging (MRI) approach is a non-invasive imaging technique that could be applied to disease theragnosis. The imaging signals of this strategy depend on the physical factors, such as proton density and relaxation time (whether T1 or T2), in the produced MR-images [113,114]. The proton differences in the water molecules predict the MR-image qualities, further allowing us to monitor the site-specific microenvironmental conditions to visually differentiate the disease progression or therapeutic advancements [115]. These image contrast capabilities could be modified using the various forms of nanomaterials via enhancing the T1 and T2 related candidates towards the improvements in the specificity for detection via MRI. Hence, in these aspects, different sizes or other physical parameters associated with colloidal, electronic, optical, and magnetic nanomaterials are emerging to improve biomedical applications [116]. Moreover, the recent signs of progress in metal nanoparticles envisage tremendous potential for biosensing, DNA hybridization, or other polymeric agent-coated materials that could be used for site-specific delivery and imaging of theragnosis [117]. Likewise, the use of MnO nanomaterials coated with functional protein candidates, such as albumin, is potentially applied for the contrast-enhanced MR-imaging of tumors [118]. In addition, the iron oxide nanomaterials with various coating or surface modifications are utilized for MR whole-body imaging in clinics [17]. Similarly, iron nanomaterial candidates showed T1 and T2 weighted images in MR-imaging applications. The gold-iron oxide hybrid nanomaterials showed promising theranostic potentials in various forms of MR-imaging functionalities in brain imaging [119].

**Figure 6:**
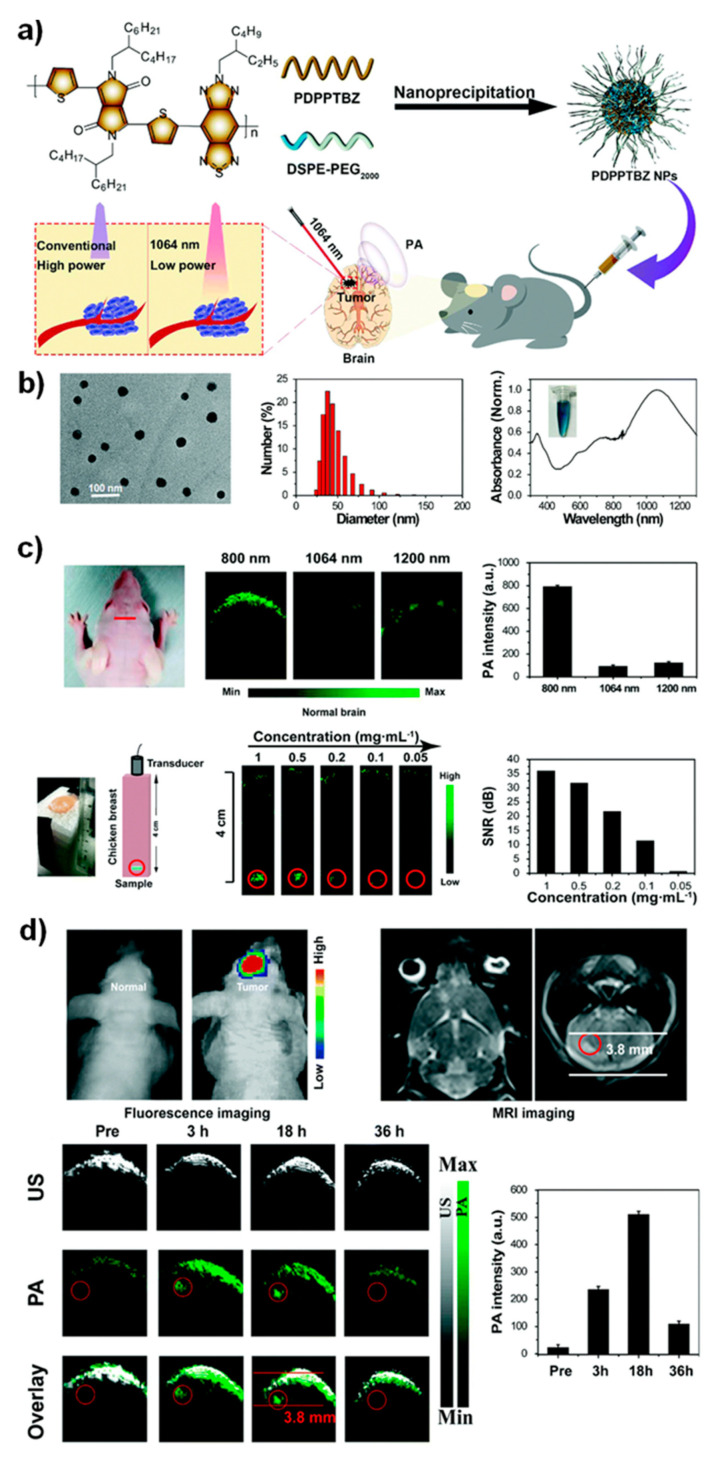
(**a**). Scheme with representative images shows the functional polymer associated PAI nanomaterials. (**b**) TEM morphology, size, and absorption (UV) features, (**c**) PAI scanning of the mice brain under the pulsed field lasers and corresponding quantification analysis, and (**d**) bioluminescence, MRI, and PA imaging conditions of the mice brain shows the ultrasound and PA signals produced by the nanoparticles with grey and green color, respectively. Reprinted with permission from Ref. [124]. Copyright 2021 Royal Society of Chemistry.

The growing interest in the application of photoacoustic imaging (PAI) modalities in clinics translates a variety of cancer surgery guidance in the surgical process. These hybrid imaging platforms of both ultrasound and light (NIR, near infrared) effects mediate the function of the photoacoustic (PA) waves in imaging which further provides the detection of acoustic wave methods that suppresses the use of optical detections [120]. Henceforth, this PAI methodologies would improve imaging signals at the deep-penetrated tissue regions via the production of the high spatial mode of resolution [43]. Via combination of various chemical and functional nanomaterials fluorophores could be used in endogenously for the high contrast image signals thereby; we can achieve the structural imaging of the brain functions through the oxygen saturations. In recent decades, the PAI applications in the clinical level are rapidly growing, necessitating the essential strategies of innovations [121]. Thus, upon employing the functional nanomaterials developmental strategies, the nanomaterials with improved imaging signals towards the generation of optical absorbance can target the diseased sites of the brain elements that could have been easily sensitized for the PAI conditions [122,123]. These functional nanomaterials, such as organic, quantum dots, functional polymer coupled agents, inorganic contrast imaging agents or nanostructures, copper and carbon nanomaterials, and small molecule coupled fluorophores with a variety of applicatory potentials, have led the potential translations of PAI in associated neurological diseases, including glioblastoma and other cancers. Yang et.al. [124] proposed a functional semiconducting polymer-based nanomaterials platform for contrast imaging applications in multimodal imaging of orthotopic brain tumors (glioblastoma) via applying the PAI with pulsed lased as a NIR light source (Figure 6). The study demonstrated the engineered nanomaterials of the report had the greater potentials of NIR and PA for deep tissue penetrated imaging by utilizing the nanoparticles as high contrast imaging candidates. Due to the nanomaterials’ maximal absorption behavior (NIR-II 1064 nm), the higher image contrast signals were produced from the deep-tissue regions of the glioma in vivo without causing any severe damage to the normal tissues. Furthermore, the glioma detections can be improved via labeling the nanomaterials with tumor-specific contrast agents; thereby, we can improve the selective functional aspects, such as sensitivity and specificity features, and efficiently achieve the site-specific tumor imaging [125]. The growth of nanomaterials is an important need in this current scenario. The development of tumor-specific exogenous imaging probe conjugates via comprising the more prolonged absorption and specificity towards PAI of brain tumors or diseases [126,127]. The functional nanomaterials in these criteria could further reduce the number of challenges associated with the PAI-associated theranostic platforms. This could improve the accurate and precise localization of delivered image contrast agents to deep brain macro/microenvironments in vivo [128,129]. Moreover, improvements in nanomaterials associated delivery, toxicity to normal tissue regions, and surround tissue absorption of ultrasound and light energies are needed to be optimized in future investigations to envisage this PAI for better improvements with accurate detection/therapeutic aspects in translational medicine.

Positron emission tomography (PET) is a functional molecular imaging platform that depends on the delivery of positron-emitting radioisotopes with commonly used atomic molecules, such as carbon (^11^C), Nitrogen (^15^N), Oxygen (^15^O), Fluorine (^18^F), ^64^Cu, and ^89^Zr labeling, on the therapeutic and imaging compounds [130]. This allows the tracing of theranostic signals via utilizing the functional chemical properties of the materials by systemic injection through the bloodstream. After the delivery, these radiotracers could metabolically target and specifically accumulate in cells, and their natural decay emits the positrons from the body tissues. The combination of PET tracers with nanomaterials offers various potential functionalities via multimodal imaging aspects. Likewise, liposomes, polymer-coated nanomaterials, and other delivery platforms offer a therapeutic radiotracer to wipe out brain tumor theragnosis [131,132]. These functionalities can be used for the imaging of brain related neurological disorders as well as biodistribution in the defected regions of pre-clinical animal models, and for the monitoring of nanoparticle delivering bioactivities [133]. To envisage the potentials of nanomaterials associated modifications in PET imaging, or a combination of PET/optical imaging, several probes are currently under the utilization.

Moreover, the optical imaging platform comprised light illumination based on electromagnetic irradiation in the ultraviolet (UV), visible, and infrared spectral regions [134]. This is the major and widely used imaging modality among the functional molecular imaging tools of characterization for visualizing the objects compared to other imaging platforms because of their target detections with higher sensitivity [135]. Cancer research utilizes optical imaging of fluorescence and bioluminescence principles to detect oncogenic/therapeutic signals in the pre-clinical models [136]. A recent study in the glioma models showed that the nanomaterials comprised of bioluminescent probes employed to monitor the disease treatment theragnosis. Similarly, bioluminescence probes could also be combined with MR-imaging using metal nanoparticles and possibly be developed for the T1 and T2 associated MR-contrast imaging and up-conversion of fluorescent contrast agents. In addition, the tissue engineering of stem cells and regenerative medicine utilize these optical imaging platforms for more comprehensive study needs in the application platforms to emphasize the role of the materials in mechanistic aspects.

Similarly, the FRET imaging molecular candidates in the magnetic nanomaterials possibly coupled to the surface modified materials enriched the properties of the signals based on magnetic fields. They improved the FRET signal intensities via energy transfer functions [137,138]. The recent research shows that developing nanomaterials with functional imaging modalities for biomedical application use various fluorescent conjugates. Similarly, the QDs have a broad range of potentials in high absorption coefficients through a more comprehensive spectral range in photostability and brightness in contrast at the elevated stages. In stem cell engineering, the QDs could modify or alter the mechanisms of cellular transport functions in those coated with ligands or peptides [139,140,141]. At the same time, the QDs were utilized to trace neural disorders and to monitor the individual synaptic receptor candidate in the neural circuits. Many reports employed the QDs as fluorescent tags for bioluminescent imaging upon several chemical or biological modifications to study the genomic aberrations, instability, cancers, and bacterial infections [46,142]. The QDs with polymer surfactants and modifiers stimulate the accumulation of materials and contrast levels in the brain sites during the in vivo administration [143,144]. Hence, the employment of functionalized nanomaterials in the brain targeting functions could improve, and the applicable strategies can push the importance towards growing theranostics fields. In addition, the utilization of nano-theranostics as multimodal imaging candidates promises significant improvements in clinical conditions, especially for cognitive disorders.

## 5. Conclusions and Future Directions

In summary, the potential growth of nanotechnology in molecular imaging with the advances in brain theranostic imaging has been evidenced by various studies, which effectively achieved early diagnoses with therapies. Even though contrast agents are traditionally used in imaging, they suffer from various adverse effects with life-threatening toxicities in patients. The enhanced properties of multifunctional futuristic nanomaterials have been liberating from these adverse effects due to their astonishing functionalities, specifically the ability to alter the delivery/imaging properties of the surrounding tissues via contrast imaging signals. On the other hand, several combined strategies with chemotherapeutic and radiotherapy agents have been applied for treating brain-related disorders without encountering any side effects. As well as in cancers, compared to the conventional treatment modalities, nanomaterials are proven to be effective as a functional nanocarrier for targeted drug delivery systems, whereby the effectiveness of the chemotherapeutic drugs or agents have been utilized to the complete target sites of the tumor microenvironments of the brain. Moreover, the surface functionalization or conjugation of ligand moieties allows the nanomaterials for active targeting, while passive targeting is also achieved. The efficient size is good enough to traverse through the leaky microvasculature in cancers and surrounding tissues. Gaining interests in nano-theranostics pave significant importance in the clinics through the acquired expertise offered by various nanomaterials with diagnostic capabilities in multimodal imaging and therapeutic platforms. Hence, nanomaterials are proven to be highly efficient in generating novel theragnosis by overcoming the existing deficiencies noted with conventional diagnostic and therapeutic platforms used for associated brain disorders. Overall, the functional nanoparticle-containing small molecule drugs are formulated internationally by the unique principles of conventional drugs. The improvement of new guidelines would be required for further scientific contribution to achieving high levels of theranostic brain imaging applications.

## Figures and Tables

**Table 1:** A list of functionalized theranostic nanoformulations developed to deliver therapeutics for brain-related diseases intranasally.

Target Disease	Nanoformulation	Model Organism	Therapeutic Outcome	Ref. No.
Parkinsons	Selegiline nanoemulsion	Rat	Intranasally administered selegiline nanoemulsion improved the behavioral activities in comparison to oral administration.	[54]
Parkinsons	Resveratrol and curcumin nanoemulsion	Sheep	Intranasal delivery of hyaluronic acid-based lipidic nanoemulsion proven as a successful carrier to enhance the solubility, stability, and brain targetability of polyphenols.	[55]
Alzheimer’s disease	Rivastigmine-loaded nanoemulsion	Rat	Achieved higher drug delivery to the brain with enhanced safety, non-toxic and non-irritating to the nasal mucosa.	[56]
Alzheimer’s disease	Donepezil nanoemulsion	Pig	Effective strategy using polymers improved the adhesion and penetration of the drug through the nasal mucosa.	[57]
Alzheimer’s disease	Cholera Toxin B subunit-based nanoparticles	Mice	Delivered nanosystem exhibited a notable performance in accumulating in the hippocampus that further showed an excellent magnetic resonance imaging (MRI) potential in vivo.	[3]
Epilepsy	Letrozole loaded nanoemulsion	Mice	Intranasal administration of nanoemulsion improved the prolonged drug release profile in brain as compared to suspension.	[58]
Migraine	Zolmitriptan mucoadhesive nanoemulsion	Rat	In vivo delivery showed higher permeability through the nasal mucosa.	[59]
Neuroprotective	Kaempferol loaded chitosan nanoemulsion	Rat	In vivo delivery and biodistribution studies exhibited a higher drug concentration in the brain upon intranasal administration.	[60]
Glioblastoma	Bevacizumab-PLGA NPs	Mice	Bevacizumab-loaded PLGA NPs showed effective tumor reductions as accompanied by higher anti-angiogenic potentials than free drug.	[61]
Glioma	Ecto-50-nucleotidase (CD73 siRNA) nanoemulsion	Rat	Intranasal nasal administration of cationic nanoemulsion with CD73 siRNA delivery system improved glioblastoma therapy.	[62]
Glioma	Temozolomide-Anti-EPHA3 PLGA NPs	Rat	Study results indicated that anti-EPHA3-decorated PLGA NPs targeted the Glioma via a nose-to-brain drug delivery approach.	[63]
Glioblastoma	Farnesylthiosalicylicacid (FTA) loaded hybrid NPs	Rat	Intranasal delivery of FTA-NPs improved the glioblastoma therapy in vivo.	[64]
Glioblastoma	miR-100 and antimiR-21 loaded PolyGIONS	Mice	Intranasal delivery of NPs strategy potentiated the nano-theranostic effects in vivo.	[51]
Glioblastoma	siRNA + TMZ loaded chitosan NPs	Mice	Intranasal delivery of nanoparticle adjuvants increase the efficiency of immune-checkpoint blockade and chemotherapy in vivo.	[65]
Glioblastoma	Self-assembled BMP4 plasmid DNA with poly(beta-amino ester) NPs	Rat	Intransally administered NPs could target brain tumors to enhance targeted therapies.	[66]
Gliobastoma	Self-assembly of MPEG-PCL-Tat with siRaf-1/ Camptothecin	Rat	Nose-to-brain delivery proved the excellent therapeutic functions for treating glioblastoma.	[67]
Glioblastoma	Extracellular vesicles (EVs) loaded with CXCR4 receptor, antimiRNA-21 and miRNA-100 biomaterials	Mice	Intranasally delivered EVs with miRNA sensitized the tumor cells to treat temozolomide, thereby improving mice’s survival rate.	[68]
Epilepsy	Carbamazepine loaded carboxymethyl chitosan nanoparticles	Mice	Enhanced drug bioavailability and brain targeting was achieved via nasal administration.	[69]
Central nervous systems disorders	Rabies Virus Glycoprotein (RVG29)-Modified PLGA Nanoparticles	Mice	Engineered nanoparticulate systems proved the viral delivery vectors to target and treat CNS via intranasal delivery.	[70]
Huntington’s disease	Chitosan nanoparticles loaded with anti-HTT siRNA	Mice	Intranasal delivery proved the promising therapeutic alternative for safe and effective which further decreases the mutant HTT expression.	[71]
Ischemic stroke	17β-estradiol (E2) loaded gelatin nanoparticles	Mice	The intranasally administered nanoparticles achieved higher delivery efficacy in vivo.	[72]
Newcastle disease and infectious bronchitis	Chitosan nanoparticles loaded with the combined attenuated live vaccine	Chicken	Intranasal adjuvant and delivery carrier made a mucosal vaccine and delivery of drugs for enhanced immune functions.	[73]
SARS-CoV-2	Receptor-binding domain (RBD) of SARS-CoV-2 spike glycoprotein loaded chitosan nanoparticles	Mice	An alternative route of intranasal vaccination mimics the natural route of SARS-CoV-2 infection and stimulates both mucosal and systemic compartments of the immune responses.	[74]
SARS-CoV2 vaccine mucosal immunization	Au-nanostar-chitosan loaded with SARS CoV-2 DNA vaccine	Mice	Intranasal administered SARS-CoV2 DNA vaccines encoded the spike protein antigen loaded nanomaterial achieved the humoral antibody responses and providing long-lasting immunity.	[75]
Respiratory infection	Chitosan Nanoparticles–Adjuvanted Chlamydia Vaccine	Mice	Intranasal adjuvants induced the humoral, mucosal, cell-mediated immunity against bacterial infections in vivo by acting as nano vaccines.	[76]

## Data Availability

Not applicable.
